# Stabilisierende Wirkungen durch Kurzarbeit

**DOI:** 10.1007/s10273-021-2850-4

**Published:** 2021-02-20

**Authors:** Toralf Pusch, Hartmut Seifert

**Affiliations:** 1 Referat Arbeitsmarktanalyse, WSI in der Hans-Böckler-Stiftung, Hans-Böckler-Straße 39, 40476 Düsseldorf, Deutschland; 2 Kaiserswertherstr. 156, 40474 Düsseldorf, Deutschland

**Keywords:** J21, J28, J68

## Abstract

In der Corona-Pandemie werden durch Kurzarbeit und weitere Hilfsmaßnahmen ganze Branchen stabilisiert. Dies gilt insbesondere für die stark betroffene Gastronomie. Kurzarbeit sichert bedrohte Arbeitsverhältnisse und dämpft Einkommenseinbußen, die bei Verlust des Arbeitsplatzes eintreten würden. Eine wesentliche Rolle spielen aufstockende Leistungen, die Kurzarbeiter:innen in tarifgebundenen Betrieben häufiger erhalten.

## Abstract (en)

During the Coronavirus pandemic, short-time work and other relief measures have stabilised various industries in Germany, particularly the hospitality sector. Short-time work safeguards not only threatened jobs but also lessens income losses that would occur if jobs were lost. Moreover, a stabilising role for the income situation and public support for crisis management policies are also favoured by income top-ups of short-time workers, especially in companies that are covered by collective agreements.

Kurzarbeitergeld spielt in der durch die Corona-Pandemie verursachten Krise eine bedeutendere Rolle zur Stabilisierung der Beschäftigung als zunächst erwartet. Hatte die Gemeinschaftsdiagnose vom Frühjahr für das zweite Quartal 2020 noch mit etwa 2,4 Mio. Beschäftigten in Kurzarbeit gerechnet (Gemeinschaftsdiagnose, 2020), so wurde dieser Wert mit knapp 5,4 Mio. bei Weitem übertroffen (Bundesagentur für Arbeit [BA], 2021). Nach zwischenzeitlicher Entspannung zur Mitte des Jahres 2020 zogen die Zahlen zur Kurzarbeit als Folge des zweiten Lockdowns zum Ende des Jahres 2020 wieder auf geschätzte 2,26 Mio. (November) an. Aus dem ursprünglich erwarteten v-förmigen ist ein w-förmiger Konjunkturverlauf geworden. Ausmaß und Dauer der Kurzarbeit sprengen mittlerweile alle aus früheren Krisenzeiten bekannten Dimensionen.[1]

Schätzungen der Bundesagentur für Arbeit (2021) beziffern die jahresdurchschnittliche Zahl der Kurzarbeitenden für 2020 auf 2,9 Mio. oder 8,7 % der sozialversicherungspflichtig Beschäftigten. Für alle Kurzarbeiter:innen wird ein durchschnittlicher Arbeitszeitausfall von etwa 38 % errechnet, der einem Beschäftigungsäquivalent bzw. einer vermiedenen Arbeitslosenzahl von ca. 1,1 Mio. entspricht. Neben Kurzarbeit tragen weitere Formen der Arbeitszeitanpassung zur Stabilisierung der Beschäftigung bei (Bellmann et al., 2020), die hier aber nicht betrachtet werden sollen.

Kurzarbeit kann Betrieben und Beschäftigten bei nachlassenden wirtschaftlichen Aktivitäten gegenüber der Alternative Entlassungen Vorteile bieten. Betriebe brauchen weder Kosten für Entlassung (Sozialpläne, Abfindungen) noch bei konjunktureller Verbesserung für Wiedereinstellung (Anwerbung, Einarbeitung) aufzubringen. Vor allem überall dort, wo Methoden agilen Arbeitens Eingang in die Arbeitsorganisation gefunden haben, kommt Teamarbeit und daraus resultierender Teamproduktivität eine hohe Bedeutung zu. Funktionsfähige Arbeitsgruppen brauchen Zeit, bis sie eingespielt sind. Und umgekehrt hat diese Form der Flexibilität auch Vorteile für Beschäftigte. Sie behalten ihre Arbeitsverhältnisse, damit die eigenständige Einkommensquelle und Ansprüche aus Seniorität oder anderen betrieblichen Leistungen. Außerdem vermeiden sie den allmählichen Verlust beruflicher Qualifikationen durch ansonsten längere Nichtbeschäftigung. Dem stehen allerdings Einkommensverluste aufgrund verkürzter Arbeitszeiten gegenüber, die jedoch bei Arbeitslosigkeit noch größer ausfallen würden. Aber auch für die öffentliche Hand kann sich Kurzarbeit gegenüber der Alternative Arbeitslosigkeit rechnen (Weber, 2020).

Vor diesem Hintergrund präsentiert der nachfolgende Beitrag Befunde aus der Erwerbspersonenerhebung der Hans-Böckler-Stiftung (HBS) vom November

1 In der ersten Ölpreiskrise 1973/1974 wurden in der Spitzenzeit etwa 700.000 Kurzarbeiter:innen oder 3,4 % der sozialversicherungspflichtig Beschäftigten gezählt, nach der deutschen Wiedervereinigung waren es 2,1 Mio. oder 7,2 % und in der Finanzkrise 2008/2009 gut 1,4 Mio. oder 5,2 %. Im bisherigen Höhepunkt, April 2020, lauteten die Vergleichswerte 6,06 Mio. oder 18 %; (BA, 2021 und verschiedene Jahrgänge).

**Dr. Toralf Pusch** leitet das Referat Arbeitsmarktanalyse am Wirtschafts- und Sozialwissenschaftlichen Institut (WSI) der Hans-Böckler-Stiftung in Düsseldorf.

**Dr. Hartmut Seifert** war bis 2009 Leiter der Abteilung WSI der Hans-Böckler-Stiftung.

2020, die einen Einblick vor allem in die soziale Lage von Kurzarbeiter:innen gewähren. Bei einigen Aspekten werden sie mit denen der beiden vorangegangenen Erhebungen vom April und Juni 2020 (Pusch und Seifert, 2020) verglichen. Die Zeitintervalle zwischen den Erhebungszeitpunkten sind zwar knapp. Es interessiert aber die Frage, inwieweit der Faktor Zeit die Einkommenssituation und auch die Bewertung der Krisenpolitik der Bundesregierung beeinflusst.

Grundlage für die empirischen Auswertungen bilden die drei Wellen der Erwerbspersonenbefragung der Hans-Böckler-Stiftung von 2020. Die Erhebungen erfolgten als Quotenstichproben im Rahmen eines Online-Access-Panels.[2] Die erste Welle wurde Mitte April in der Phase des Corona-Lockdowns erhoben und umfasst Daten von 7.677 Erwerbspersonen. Für die zweite und dritte Welle, die Ende Juni und Anfang November erhoben wurden, wurde keine Panelauffrischung vorgenommen. Mit immerhin 6.309 bzw. 6.102 befragten Erwerbspersonen blieb der Panelabrieb allerdings verhältnismäßig klein.

## Wer arbeitet kurz?

Der infolge der Corona-Pandemie verhängte Lockdown trifft Betriebe und Wirtschaftszweige sehr unterschiedlich. Ein Teil ist von Geschäftsschließungen und Nachfrageeinbrüchen betroffen, ein anderer hat zusätzliche Leistungen zu erbringen und den Arbeitseinsatz aufzustocken. Hierauf können Betriebe entweder mit der Variation der Arbeitszeit und/oder der Beschäftigtenzahl reagieren. Die weitere Aufmerksamkeit gilt der ersten Variante.

Besonders einschneidend wirken sich die Kontakt- und Hygienemaßnahmen auf Teile des Dienstleistungsgewerbes, vor allem das Gastgewerbe aber auch das Verarbeitende Gewerbe aus. Im Gastgewerbe hatten Anfang November nahezu zwei Drittel (65,4 %) der Beschäftigten die Arbeitszeit reduziert, knapp die Hälfte der sozialversicherungspflichtig Beschäftigten arbeitet kurz (vgl. Abbildung 1). Im Sommer ging der Anteil der Kurzarbeit im Gastgewerbe zurück, worauf die geringere durchschnittliche Dauer der Kurzarbeit (4,4 Monate zum Befragungspunkt Anfang November) im Vergleich zum Rest der Wirtschaft (5,9 Monate) hindeutet.

Abbildung 1
**Kurzarbeit nach Branchen, November 2020**
Beschäftigtenquoten in %

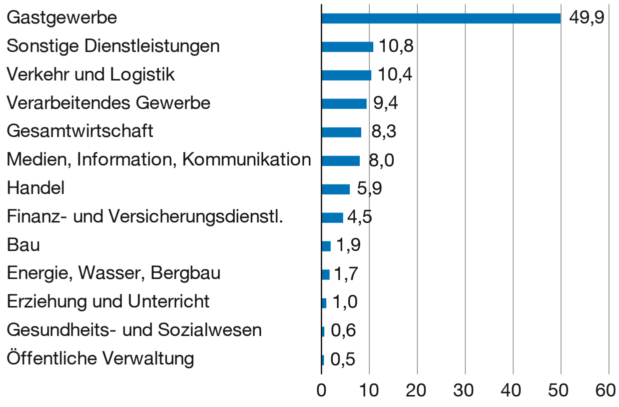

Quelle: HBS Erwerbspersonenbefragung; eigene Berechnungen.

Auch in anderen Branchen hat sich der Umfang der Kurzarbeit im Jahresverlauf geändert, wie ein Abgleich mit den Daten der Juni-Erhebung der Erwerbspersonenbefragung zeigt (Pusch und Seifert, 2020). So war die Quote der Kurzarbeit im Verarbeitenden Gewerbe im Juni mit 20 % noch deutlich über dem Durchschnittswert von 13 %, im November 2020 nur noch leicht überdurchschnittlich (9,4 %). Auch in der Arbeitnehmerüberlassung hat sich die Quote auf 5,9 % halbiert. Erstmals seit März 2020 ist diese Beschäftigtengruppe anspruchsberechtigt. Gerade in diesem ansonsten von wenig stabiler Beschäftigung gekennzeichneten Bereich kann Kurzarbeit zunächst gefährdete Beschäftigungsverhältnisse sichern. Die Verlängerung der Bezugsdauer hat aber offensichtlich nur einem Teil der Beschäftigten bislang helfen können, die unsichere Arbeitsmarktlage zu überbrücken. Denn im Oktober 2020 war die Zahl der Beschäftigten in Leiharbeit bereits um 10,4 % gegenüber dem Vorjahreswert geschrumpft (BA, 2021).

Nicht vom Schutzschirm der Kurzarbeit abgesichert sind dagegen Minijobber:innen. Deren Zahl ist im Oktober um 304.000 oder 6,7 % unter den Vorjahreswert gesunken (BA, 2021). Vermutlich dürfte vor allem das Gastgewerbe, das insgesamt im September 6,5 % weniger Beschäftigte zählte, einen Teil der Minijobber:innen entlassen haben, die in diesem Wirtschaftszweig im Vergleich zur Gesamtwirtschaft überdurchschnittlich vertreten waren.[3]

Angesichts der starken Nutzung von Kurzarbeit in Teilbereichen des Dienstleistungssektors überrascht es nicht, dass für die Wirtschaft insgesamt die Quote der Frauen

2 Dabei wurde die strukturelle Zusammensetzung der Befragten anhand von festgelegten Quoten nach den Merkmalen Alter, Geschlecht, Bundesland und Bildung abgebildet und zusätzlich mit Gewichten nachträglich korrigiert. Die Quotenvorgaben basieren auf Soll-Zahlen aus der amtlichen Statistik.

3 Die Zahl der Minijobber:innen lag Mitte 2019 mit 1,056 Mio. nur unwesentlich unter der der sozialversicherungspflichtig Beschäftigten mit 1,109 Mio. (DEHOGA, 2020).

Abbildung 2
**Kurzarbeit nach Betriebsgröße, November 2020**
Quoten in %

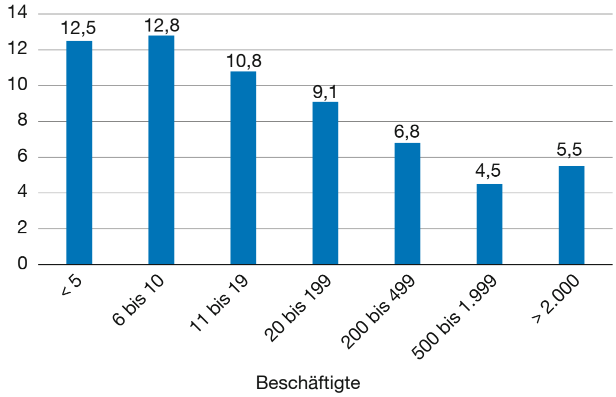

Quelle: HBS Erwerbspersonenbefragung; eigene Berechnungen.

Abbildung 3
**Aufstockende Leistungen, Juni und November 2020**
Quoten in %

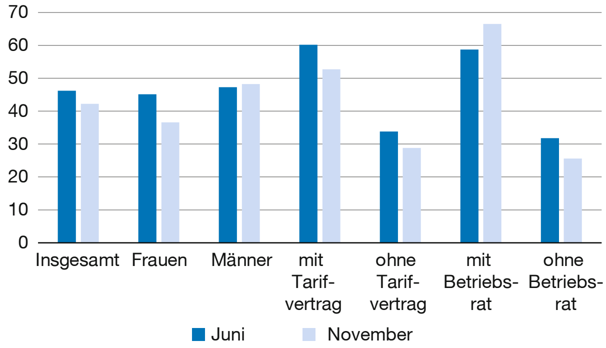

Quelle: HBS Erwerbspersonenbefragung; eigene Berechnungen.

in Kurzarbeit (9 %) etwas höher ist als die der Männer (7,6 %). Genau umgekehrt lauteten demgegenüber in der Finanzkrise 2008/2009 wegen des starken Konjunktureinbruchs im Verarbeitenden Gewerbe die Vergleichswerte 2,3 % für Frauen und 6,3 % für Männer.

Stark unterscheidet sich die Nutzung des Kurzarbeitergelds nach der Betriebsgröße. In Klein- und Kleinstbetrieben ist der Anteil der Kurzarbeiter:innen deutlich höher als in größeren (vgl. Abbildung 2). Die hohen Anteilswerte hängen vor allem mit dem kleinbetrieblich organisierten Gastgewerbe zusammen. Gerade in dieser Betriebsgrößenklasse übernimmt Kurzarbeit eine wichtige beschäftigungssichernde Funktion, da der gesetzliche Kündigungsschutz erst in Betrieben mit mehr als zehn Beschäftigten greift.

## Aufstockende Leistungen

Das Kurzarbeitergeld ersetzt 60 % bzw. 67 % des Nettoentgelts der ausgefallenen Arbeitszeit (SGB III, § 96). Im Unterschied zu früheren Krisen hat die Bundesregierung jedoch bald nach Beginn der Corona-Pandemie bereits im März 2020 die Höhe des Kurzarbeitergelds ab dem vierten Monat der Bezugsdauer auf 70 % bzw. 77 % und ab dem siebenten Monat auf 80 % bzw. 87 % angehoben (Bundesministerium für Arbeit und Soziales [BMAS], 2020). Außerdem sehen zahlreiche Tarifverträge erhöhte Leistungen zum Kurzarbeitergeld vor und federn so die Einkommensverluste bereits von Beginn an ab (Schulten und WSI-Tarifarchiv, 2020). Gerade in den unteren Einkommensklassen kann Kurzarbeit bereits früh zu sozialen Härten führen, da hier finanzielle Reserven selten sind, häufig die Sparquote sogar negativ ist (Späth und Schmid, 2016) und deshalb vermutlich kaum Spielraum für eine weitere Schuldenaufnahme besteht. Je länger Kurzarbeit andauert, desto erforderlicher werden soziale Transferleistungen. Umso wichtiger sind deshalb tarifliche und betriebliche Vereinbarungen über aufstockende Leistungen.

Nicht alle Beschäftigten in Kurzarbeit profitieren jedoch von diesen zusätzlichen Zahlungen. Nicht ganz die Hälfte berichtet über ein erhöhtes Kurzarbeitergeld[4] (vgl. Abbildung 3). Im Vergleich der beiden Erhebungszeitpunkte Juni und November ist der Anteil der Kurzarbeiter:innen mit aufstockenden Leistungen etwas zurückgegangen. Diese Entwicklung dürfte vorrangig mit dem Rückgang der Kurzarbeitszahlen im Verarbeitenden Gewerbe zu tun haben, wo die Tarifbindung und damit die Verbreitung von aufstockenden Regelungen höher als in den Dienstleistungssektoren ist. Gute Chancen auf relativ geringe Einkommensverluste haben Kurzarbeiter:innen in Betrieben mit Betriebsrat oder Tarifbindung. Inwieweit die erhöhten Leistungen auch auf die verbesserten gesetzlichen Regelungen zurückzuführen sind, lässt sich mit den vorliegenden Daten nicht sagen, es könnte aber vermutlich bei den Juni- und Novemberwerten teilweise der Fall sein.[5]

Die Chancen, als Kurzarbeiter:in aufstockende Leistungen zu beziehen, hängen stark von der Branchenzugehörigkeit ab (vgl. Abbildung 4). Wer in der Finanz- und Ver-

4 Knapp die Hälfte der Beschäftigten arbeitet in Betrieben mit Tarifbindung (Ellguth und Kohaut, 2020).
5 Da aus Tarifverträgen begründete aufstockende Leistungen mit gesetzlichen Leistungen verrechnet werden, ist es für Beschäftigte nicht genau zu erkennen, auf welchen Anspruchsgrundlagen die Leistungen basieren.

Abbildung 4

**Quoten der Aufstocker an den Kurzarbeiter:innen nach Branchen[1], November 2020**

in %

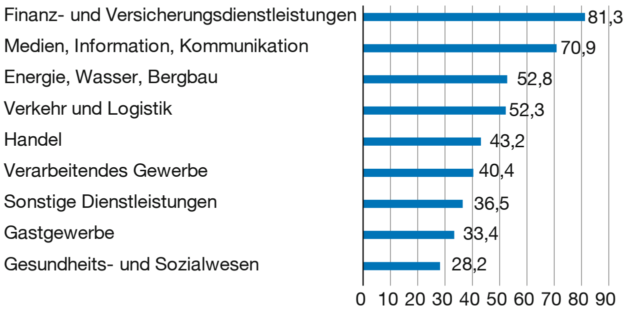

[1] Branchen mit geringer Fallzahl und Aufstocker-Quote 0 wurden nicht abgebildet.

Quelle: HBS Erwerbspersonenbefragung; eigene Berechnungen.

Abbildung 5

**Individuelles Nettoeinkommen, November 2020**

in Euro

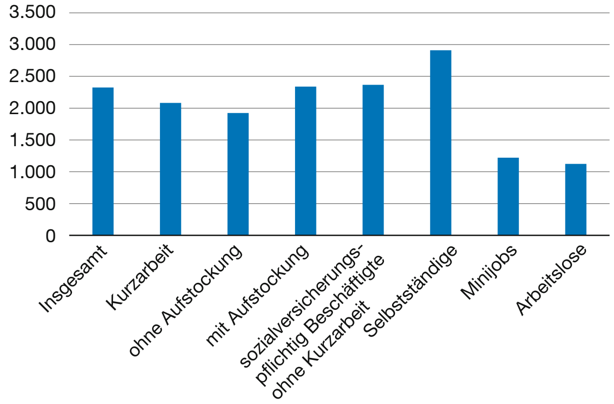

Anmerkung: Die Einkommen wurden mit den mittleren Werten der abgefragten Einkommensklassen approximiert.

Quelle: HBS Erwerbspersonenbefragung; eigene Berechnungen.

sicherungswirtschaft arbeitet, hat deutlich höhere Chancen, mehr als das gesetzlich vorgeschriebene Ausfallgeld zu beziehen als im Gastgewerbe oder in den sonstigen Dienstleistungen. Besonders groß fallen die Unterschiede im Verarbeitenden Gewerbe aus; in tarifgebundenen Betrieben erhalten 62 % der Beschäftigten ein erhöhtes Kurzarbeitergeld in den tariffreien dagegen nur 11,6 %.

Auch die Höhe der aufstockenden Leistungen differiert stark. Etwa die Hälfte der Kurzarbeiter:innen mit erhöhtem Kurzarbeitergeld erhält Leistungen bis zu 80 % des ausgefallenen Nettoentgelts und nur ein kleiner Anteil sogar über 90 %. In tarifgebundenen Betrieben ist die Wahrscheinlichkeit auf ein aufgestocktes Kurzarbeitergeld zwischen 80 % und 90 % höher als in den tariffreien, wobei in Rechnung zu stellen ist, dass in letzteren relativ weniger Kurzarbeiter:innen überhaupt zusätzliche Zahlungen erhalten.

## Einkommen und finanzielle Belastungen

Die wirtschaftlichen Folgen der Corona-Pandemie gehen nicht spurlos an den Beschäftigten vorbei, treffen diese aber sehr unterschiedlich. So ist das individuelle monatliche Nettoeinkommen der Kurzarbeiter:innen im Vergleich zu den übrigen sozialversicherungspflichtig Beschäftigten deutlich niedriger, was allerdings zum Teil auch auf strukturelle Faktoren wie die Branchenzusammensetzung der Kurzarbeit zurückzuführen ist. Besonders groß fällt der Abstand mit ca. 400 Euro bei den Kurzarbeiter:innen ohne aufstockende Leistungen aus (vgl. Abbildung 5). Deutlich kleiner ist er auch hier bei Kurzarbeiter:innen mit aufstockenden Leistungen, was auch darauf zurückzuführen sein dürfte, dass diese häufiger in tarifgebundenen Betrieben arbeiten. Verdienste von Beschäftigten in tarifgebundenen Betrieben sind im Schnitt höher als solche in Betrieben ohne Tarifbindung (Lübker und Schulten, 2019).

Um dieses und weitere Strukturmerkmale zu berücksichtigen, wurde zusätzlich eine Regressionsanalyse für die Einflussfaktoren der Einkommen von sozialversicherungspflichtig Beschäftigten durchgeführt. Dabei konnte ein positiver Einfluss der Tarifbindung des Verdienstes auf die Einkommen und ein negativer Einfluss von Kurzarbeit sowie eine deutliche Dämpfung des Einkommensverlusts durch aufstockende Leistungen bestätigt werden.[6]

Während nur ein kleiner Teil der sozialversicherungspflichtig Beschäftigten ohne Kurzarbeit die finanzielle Situation als stark belastend empfindet, ist es bei den Kurzarbeiter:innen ohne zusätzliche Leistungen mehr als die Hälfte, deutlich weniger sind es dagegen bei denjenigen mit solchen Leistungen (vgl. Abbildung 6). Gleichwohl empfindet selbst in dieser Gruppe ein beträchtlicher Anteil der Personen die finanziellen Einbußen als stark belastend. Fasst man die beiden Kategorien „kaum" und „gar nicht" bei der Frage nach den finanziellen Be-

6 Regressionsergebnisse auf Anfrage erhältlich. Die Regressionen wurden angelehnt an Lohnregressionen als *ordered logistic regression* durchgeführt, mit den Klassendaten für die Individualeinkommen sowie als lineare Regression mit approximierten Einkommen (Klassenmitte), um die Größenordnung der Effekte abschätzen zu können. Kontrolliert wurde für soziodemografische Faktoren, Charakteristiken der Beschäftigung (Branchen, Stellung im Beruf), Qualifikation und eine approximierte Dauer der Beschäftigung (Alter – 16).

Abbildung 6
**Grad der Belastung durch finanzielle Situation, November 2020**

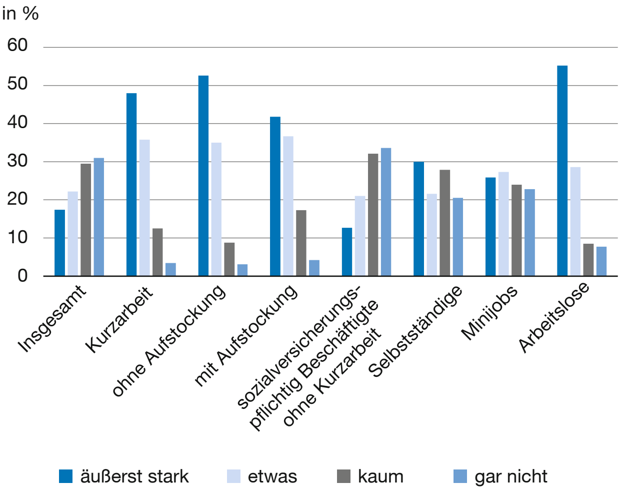

Quelle: HBS Erwerbspersonenbefragung; eigene Berechnungen.

Abbildung 7
**Furcht vor Arbeitslosigkeit/Erwerbslosigkeit[1], November 2020**

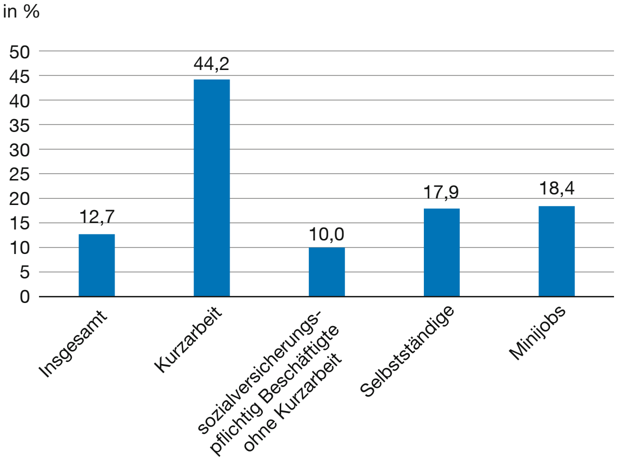

[1] Anteile der Antworten „ja, auf jeden Fall“ oder „eher ja“.

Quelle: HBS Erwerbspersonenbefragung; eigene Berechnungen.

lastungen zusammen, dann empfinden zwei Drittel der nicht-kurzarbeitenden sozialversicherungspflichtig Beschäftigten die finanzielle Situation grosso modo als nicht belastend, aber mit 12 % nur ein sehr kleiner Anteil der Kurzarbeitenden ohne zusätzliche Leistungen – ein überraschungsfreies Ergebnis. Zusätzlich durchgeführte Regressionsanalysen konnten einen mildernden Einfluss der aufstockenden Regelungen auf die finanzielle Belastungssituation zeigen. Zudem fällt die finanzielle Belastung bei Kurzarbeiter:innen geringer aus als bei Beschäftigten, die seit Beginn der Corona-Pandemie ihren Arbeitsplatz verloren haben.[7]

## Furcht vor Verlust der Beschäftigung

Die Furcht vor Arbeitslosigkeit ist unter Kurzarbeiter:innen wesentlich weiter verbreitet als unter den übrigen Erwerbstätigen. Aber immerhin mehr als die Hälfte der Kurzarbeiter:innen äußert keine oder nur geringe Angst vor einem Jobverlust und geht offensichtlich davon aus, durch Kurzarbeit das Beschäftigungsverhältnis sichern zu können (vgl. Abbildung 7).[8] Ohne Arbeit zu sein, wird auch von Selbstständigen überdurchschnittlich häufig als Bedrohung gesehen. Überdurchschnittlich verbreitet sind Sorgen um den Arbeitsplatz in Branchen mit einer hohen Kurzarbeiterquote. Während in der Gesamtwirtschaft ca. 12,7 % der Erwerbstätigen um ihren Arbeitsplatz fürchten, sind es im Gastgewerbe 35 %, im Verarbeitenden Gewerbe 14,6 % und im Handel 15,7 % der Erwerbstätigen. Offensichtlich deutet sich hier bereits der durch die Corona-Pandemie verstärkte Strukturwandel in Richtung zu mehr Online-Handel an.

Ein ähnliches Bild bei der Häufigkeitsverteilung für die hier betrachteten Erwerbsgruppen findet man auch bei den Antworten auf die Frage nach Ängsten um die eigene wirtschaftliche Existenz. Die Sorge um den Job ist nicht zu trennen von der Sorge darüber, wie es wirtschaftlich weitergehen wird. Dieser Zusammenhang zeigt sich bei 85 % von denen, die um ihren Job fürchten, aber nur bei gut 10 % derjenigen ohne Jobangst. Während etwas mehr als die Hälfte der Kurzarbeiter:innen Existenzängste zeigt, trifft das nur auf etwa jeden Sechsten der sozialversicherungspflichtig Beschäftigten ohne Kurzarbeit zu, aber auf fast jeden dritten Selbstständigen. Ein zumindest als gesichert geglaubter Arbeitsplatz befreit offensichtlich von materiellen Zukunftssorgen.

Nicht überraschend ist, dass unter denjenigen Personengruppen mit vermutlich eher als ungesichert angesehenen Beschäftigungsverhältnissen und im Schnitt geringeren finanziellen Rücklagen die Existenzängste besonders verbreitet sind. Das ist bei Kurzarbeiter:innen eher der Fall als bei Beschäftigten ohne Kurzarbeit. In der erstgenannten Gruppe gibt fast die Hälfte an, nicht über ausreichende Rücklagen zu verfügen, bei denen ohne aufstockende Leistungen sogar die Hälfte; bei den Beschäftigten ohne Kurzarbeit ist es dagegen nur etwas mehr als ein Drittel.

7 Ergebnisse erhältlich auf Anfrage. Die Regressionen wurden analog zu der Regression zur Einkommenssituation als *ordered logistic regression* durchgeführt.
8 Unbekannt ist, ob auf betrieblicher Ebene Vereinbarungen über Beschäftigungssicherungen getroffen wurden, die das Antwortverhalten beeinflussen dürften.

Abbildung 8

**Rückgriff auf Ersparnisse seit Corona-Krise, November 2020**

Anteile in %

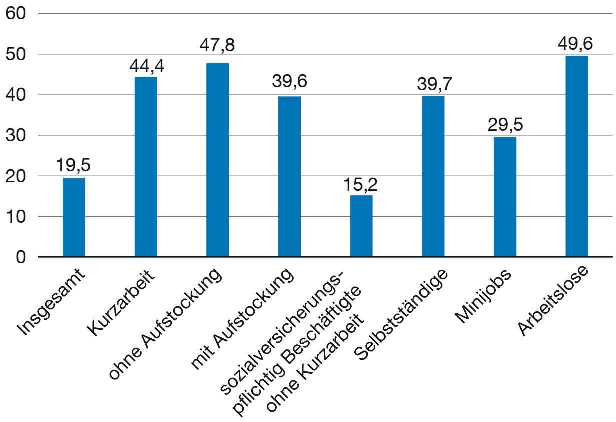

Quelle: HBS Erwerbspersonenbefragung; eigene Berechnungen.

Abbildung 9

**Zufriedenheit mit Krisenpolitik der Bundesregierung, April, Juni, November 2020**

Anteile in %

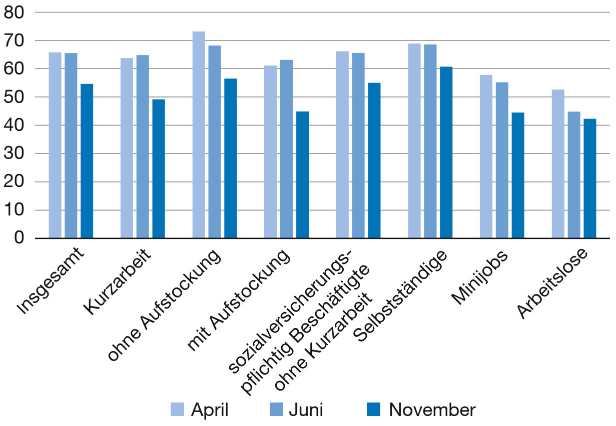

Quelle: HBS Erwerbspersonenbefragung; eigene Berechnungen.

Vor diesem Hintergrund erscheint es auch plausibel, dass seit Beginn der Corona-Krise eher Kurzarbeiter ohne Aufstockungen (47,8 %), also mit vergleichsweise großen Abstrichen gegenüber dem ehemaligen Einkommen, auf Ersparnisse zurückgegriffen haben, sofern sie über solche verfügten (vgl. Abbildung 8). Bei sozialversicherungspflichtig Beschäftigten ohne Kurzarbeit ist das deutlich seltener der Fall (15,2 %). Vor dem Hintergrund der geringeren Einkommen und Rücklagen ist auch eine Verringerung des Konsums bei den Kurzarbeiter:innen zu erwarten. Eine hierfür durchgeführte Regression konnte diesen Zusammenhang für die Kurzarbeiter:innen zeigen. Der Rückgang des Konsums fällt bei diesen geringer aus als bei den im Laufe der Pandemie in Arbeitslosigkeit gewechselten Beschäftigten.[9]

## Bewertungen Krisenpolitik

Die Zufriedenheit mit der Krisenpolitik nimmt bei allen hier betrachteten Gruppen im Zeitraum zwischen April und November 2020 ab. Dabei zeigen sich starke Unterschiede zwischen den Gruppen der Erwerbstätigen. Die höchsten Zustimmungswerte kommen von den Selbstständigen, von denen auch im November deutlich mehr als die Hälfte der Bundesregierung ein gutes Zeugnis für ihre Krisenpolitik ausstellt. Skeptischer fallen dagegen die Urteile von Arbeitslosen aus. Nur noch etwas über 40 % von ihnen bewerten die Politik positiv, nachdem es im April noch über 50 % waren (vgl. Abbildung 9). Die Frage nach der Zufriedenheit mit der Krisenpolitik der Bundesregierung ist sehr allgemein formuliert und schließt sowohl die Aktivitäten zur Eindämmung der Pandemie als auch der wirtschaftlichen Folgen ein. Spezifischer ist die Frage nach der Zufriedenheit mit Hilfsmaßnahmen zur Unterstützung von Wirtschaft und Arbeitsplätzen. Die Zustimmung fällt weniger positiv aus, nicht einmal die Hälfte der Erwerbspersonen zeigt sich zufrieden; die relativ höchsten Werte verzeichnen Kurzarbeiter:innen mit aufstockenden Leistungen mit einem deutlichen Abstand zu denen ohne solche Leistungen und zu den Minijobber:innen (vgl. Abbildung 10). Zusätzliche Leistungen, seien sie gesetzlich oder tariflich begründet, finden offensichtlich Anerkennung, hohe Beschäftigungsrisiken und fehlende soziale Absicherung wie bei den Minijobber:innen oder Arbeitslosigkeit schlagen sich dagegen in negativen Bewertungen nieder.

Auffallend sind die im Juni mit Ausnahme der Beschäftigten in Kurzarbeit ohne aufstockende Leistungen bei allen übrigen betrachteten Gruppen gegenüber April höheren Zustimmungswerte. Vermutlich haben die aufgehobenen Kontaktbeschränkungen die Stimmung aufgehellt.

## Fazit

Durch Kurzarbeit und andere Hilfsmaßnahmen werden in der Corona-Pandemie ganze Branchen stabilisiert.

9 Durchführung analog zur Regression für die Einkommensklassen als *ordered logistic regression* mit Kontrollvariablen für die Soziodemografie und Beschäftigungssituation. Ergebnisse auf Anfrage erhältlich.

Abbildung 10

**Zustimmung für Hilfsmaßnahmen[1], April, Juni, November 2020**

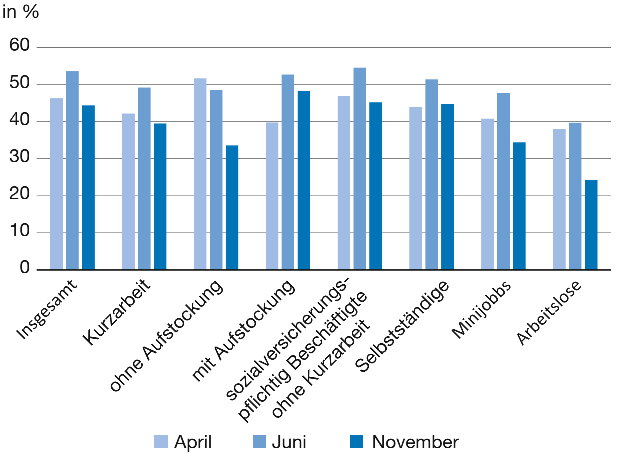

[1] Hilfsmaßnahmen zur Unterstützung der Wirtschaft und dem Erhalt von Arbeitsplätzen; Anteil der Antworten „ausreichend", ohne Angaben „weiß nicht".

Quelle: HBS Erwerbspersonenbefragung; eigene Berechnungen.

Am augenfälligsten ist dies in der stark von Kurzarbeit betroffenen Gastronomie. Kurzarbeit sichert (zunächst) nicht nur bedrohte Arbeitsverhältnisse. Sie dämpft vor allem die Einkommenseinbußen, die ansonsten im Fall von Arbeitslosigkeit eintreten würden. Eine wesentliche Rolle dabei spielen aufstockende Leistungen, die jedoch Kurzarbeiter:innen in tariffreien Betrieben seltener als in tarifgebundenen erhalten. Umso wichtiger ist vor allem mit zunehmender Dauer der Kurzarbeit der erhöhte Bezug des Kurzarbeitergelds. Kurzarbeiter:innen haben zwar im Vergleich zu Arbeitslosen seltener mit finanziellen Schwierigkeiten zu kämpfen. Gleichwohl ist auch bei ihnen mit sich im Laufe der Zeit verschärfenden sozialen Problemen zu rechnen, da finanzielle Rücklagen vielfach bereits aufgebraucht sind und bei immer mehr Personen weiter schrumpfen werden.

Einkommensstabilisierende Leistungen sowie der Erhalt des Arbeitsplatzes dürften wesentlich dazu beitragen, dass Beschäftigte in Kurzarbeit die Krisenpolitik der Bundesregierung positiver bewerten als Arbeitslose. Die Zustimmungswerte zeigen aber Erosionstendenzen. Dieser Prozess könnte sich fortsetzen oder gar beschleunigen, wenn es Kurzarbeit immer weniger gelingt, die Krisenphase zu überbrücken. Bereits im November war die Furcht vor Jobverlusten unter Kurzarbeiter:innen gerade in den besonders bedrohten kleinbetrieblichen Teilbereichen des Handels und vor allem dem Gastgewerbe stark verbreitet.
